# Transcriptional Changes in the Xylose Operon in *Bacillus licheniformis* and Their Use in Fermentation Optimization

**DOI:** 10.3390/ijms20184615

**Published:** 2019-09-18

**Authors:** Youran Li, Xiang Liu, Liang Zhang, Zhongyang Ding, Sha Xu, Zhenghua Gu, Guiyang Shi

**Affiliations:** 1 Key Laboratory of Industrial Biotechnology, Ministry of Education, Jiangnan University, Wuxi 214122, China; liyouran@jiangnan.edu.cn (Y.L.); 612277www@163.com (X.L.); zhangl@jiangnan.edu.cn (L.Z.); zyding@jiangnan.edu.cn (Z.D.); xusha1984@jiangnan.edu.cn (S.X.); guzhenghua2011@163.com (Z.G.); 2 National Engineering Laboratory for Cereal Fermentation Technology, Jiangnan University, 1800 Lihu Avenue, Wuxi 214122, China; 3 Jiangsu Provincial Research Center for Bioactive Product Processing Technology, Jiangnan University, 1800 Lihu Avenue, Wuxi 214122, China

**Keywords:** *Bacillus licheniformis*, transcriptional changes, xylose operon, fermentation optimization, expression element

## Abstract

The xylose operon is an efficient biological element used for the regulation of gene expression in *Bacillus licheniformis*. Although the mechanism underlying the xylose-mediated regulation of this operon has been elucidated, the transcriptional changes that occur under various fermentation conditions remain unclear. In this study, the effects of different conditions on xylose operon expression were investigated. Significant upregulation was observed during the transition from the logarithmic phase to the stationary phase (2.5-fold, *n* = 3, *p* < 0.01). Glucose suppressed transcription over 168-fold (*n* = 3, *p* < 0.01). Meanwhile, the inhibitory effect of glucose hardly strengthened at concentrations from 20 to 180 g/L. Furthermore, the transcription of the xylose operon increased at elevated temperatures (25–42 °C) and was optimal at a neutral pH (pH 6.5–7.0). Based on these findings, relevant fermentation strategies (delaying the induction time, using dextrin as a carbon source, increasing the fermentation temperature, and maintaining a neutral pH) were proposed. Subsequently, these strategies were validated through the use of maltogenic amylase as a reporter protein, as an 8-fold (*n* = 3, *p* < 0.01) increase in recombinant enzyme activity compared to that under unoptimized conditions was observed. This work contributes to the development of fermentation optimization and furthers the use of the xylose operon as an efficient expression element.

## 1. Introduction

*Bacillus licheniformis* is a generally recognized as safe (GRAS) workhorse that has simple culture requirements and abundant protein secretion. It has been widely used in industry for the production of endogenous enzymes, including protease, amylase, and phytases, almost without exception, via a constitutive expression pattern [1,2,3,4]. That pattern, however, is inconvenient when applied to produce certain protein products. For example, if phospholipase catalyzes the hydrolysis of cell wall lipids, the constitutive expression of phospholipase would badly hamper the proliferation of bacterial cells. Even if the target product was innocuous to cells, the overexpression of the encoding gene would put a heavy burden on the growth at the beginning of the fermentation, which would dramatically prolong the production time. Inducible expression, however, can harmonize cell growth and product synthesis to the maximum extent. This expression pattern has unique advantages in improving production performance and expanding the application range of strains [5,6]. Among the few inducible expression systems that are currently used in *Bacillus* strains, the xylose-inducible system is the earliest and most widely studied [7,8,9]. However, research on this system has mainly focused on the on–off mechanism and the related sugar signaling pathway rather than on the characteristics with respect to application.

In *B. licheniformis*, the xylose-inducible expression system has four components: *xylR* (encoding a repressor protein), *P_xyl_* (encoding the promoter of xylose isomerase), *xylA* (encoding a xylose isomerase), and *xylB* (encoding a xylulose kinase) [10]. Similar to most operons, the repressor protein is separated from complexes with the xylose repressor and binds xylose when the sugar is present in the medium. Then, the *xylA* gene, which is regulated by the xylose operon, can be expressed. In contrast, the repressor protein tightly binds to the regulatory protein and blocks *xylA* gene expression when xylose is absent [11,12]. After induction with 0.50% xylose, the expression of the reporter genes can be increased 130–350-fold in *B. megaterium*. Additionally, the expression of the xylose operon is regulated by glucose-mediated carbon catabolite repression (CCR), indicating that *B. licheniformis* preferentially utilizes glucose during fermentation when both glucose and xylose are available [13]. Nevertheless, previous studies have generally not investigated the dynamic transcriptional changes in the xylose operon during the fermentation process. Thus, the changing trends and factors that impact this expression system during the fermentation process remain unclear. As a result, it is difficult to generate a process control strategy accordingly. Currently, the optimization of the inducible expression process focuses merely on product yield; however, it is difficult to gain insights into the mechanisms of microbial gene expression through this approach [14]. This product-oriented strategy can be helpless when the target product is difficult to assay. As the function of an operon can be fine-tuned by growth stage, glucose stress level, temperature, or pH, the dynamic regulatory characteristics of an operon can indicate the generation of the product on the most essential level. More importantly, this fine tuning can serve as a reliable basis for process optimization [15]. In this sense, it is of crucial importance to better understand the expression characteristics of the xylose operon to further develop the industrial application of *B. licheniformis*.

Information on transcriptional activity is the most direct reflection of operon function. In previous studies, the transcriptional activity of the xylose operon was primarily assessed by Western blotting [7,12], which is a complicated approach only for qualitative analysis. Real-time quantitative PCR (RT-qPCR) is an important technique for detecting gene expression and is distinguished from other methods in terms of its accuracy, sensitivity, and speed of obtaining results. Because of these advantages, its applications in fermentation have greatly increased in recent years. It has been applied to trace target gene expression or to determine plasmid copy number in certain microorganisms [16,17]. Transcription-based methods can be used for in-depth quantification of gene activity, which in turn can lead to the development of rational approaches to improve productivity [18,19]. Thus, it is promising to use RT-qPCR to investigate changes in the xylose operon at the transcriptional level.

In this study, RT-qPCR was used to assess the transcriptional changes in the xylose operon in *B. licheniformis* at different growth stages, glucose stress levels, temperatures, and pH values. The results revealed characteristics of the xylose operon with respect to the regulation of target gene expression during various fermentation processes and provided a scientific reference for fermentation optimization. Then, characteristic-derived fermentation strategies were proposed to optimize the process of xylose-inducible fermentation. The gene encoding maltogenic amylase was placed under the control of the xylose operon in *B. licheniformis* to further validate the chosen strategies. This study is useful when considering the development of the operon as an efficient expression element. The approach described in this study also opens up new avenues for the optimization of fermentation conditions.

## 2. Results and Discussion

### 2.1. Establishment of an RT-qPCR-Based Detection Method in B. licheniformis

**Figure 1:**
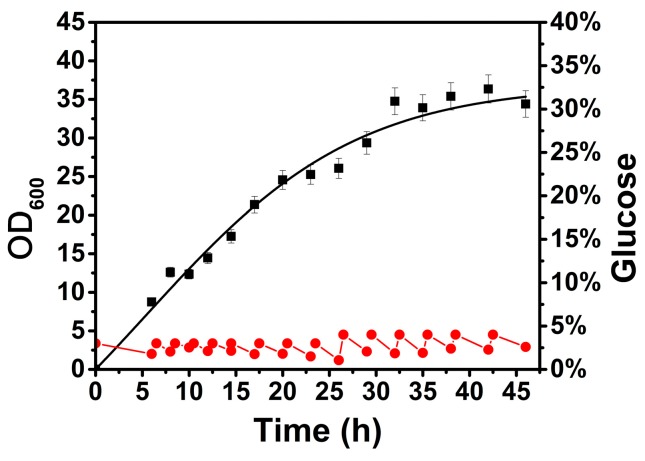
Growth and glucose consumption curve of *B. licheniformis* under basal culture conditions, where the growth curve was obtained using a nonlinear fitting. ■—■, OD_600_; ●—●, glucose concentration.

The growth and metabolism of *B. licheniformis* exhibit acute fluctuation with changes in glucose concentration [20,21]. To minimize the above impact on the transcription of the target gene, the glucose concentration in the medium was maintained at a specific level during the fermentation process, which was defined as the basal culture condition for further investigation of other influencing factors. As shown in Figure 1, *B. licheniformis* grew stably with confined glucose feeding compared to that without glucose supplementation (shown in Supplementary Material), and the biomass (OD_600_) reached approximately 35 at the stationary phase. The glucose concentration was precisely controlled at approximately 20 g/L (as detected by HPLC) by feeding at intervals. During this process, glucose was consumed at approximately 5–10 g/L per hour, and the total consumption reached 207.5 g/L. A previous study also showed that glucose could be maintained from 10 to 20 g/L in favor of the steady growth and metabolism of *B. licheniformis* [22]. Therefore, this result provides a favorable condition for investigation of the influence on gene transcription caused by a certain environmental factor in a dynamic fermentation process.

A reliable housekeeping gene as an internal control is always important for quantitative RT-PCR. The *rpsE* gene encoding a ribosomal protein has previously been used as an ideal internal control for *Bacillus* species grown at various growth stages and under various stress conditions and has exhibited constant expression levels [23,24]. Since the model strain *B. licheniformis* ATCC 9945a has not yet been reported as a reliable housekeeping gene, the *rpsE* gene from *Bacillus subtilis* could serve as a template for identifying its putative allele in the published genomic sequence of 9945a [25]. After the total RNA was extracted (supplementary data, Figure S1), a linear fit was generated using data obtained from RT-qPCR, and the following equations were obtained for standard curves: *rpsE*, y = −3.337x + 15.15 *R*^2^ = 0.9991, *E* = 98.03%; *xylA*, y = −3.186x + 15.398 *R*^2^ = 0.9979, *E* = 106.0%. The amplification efficiencies of both the *rpsE* and *xylA* genes were close to 100%, and the Ct values were linearly correlated with the different concentrations of template. Thus, the relative transcriptional activity was calculated using the 2^−ΔΔ*C*t^ method. More importantly, as the xylose isomerase encoded by *xylA* was observed to be unique in the genome of the model strain, according to the results of a homologous protein alignment, the transcription of the *xylA* using *rpsE* as a reference would directly reflect the transcriptional activity of the xylose operon, which is tightly regulated by xylose. Thus, further changes in the transcription of the xylose operon during fermentation could be studied on the basis of these findings.

### 2.2. Transcriptional Activity of the Xylose Operon at Different Growth Stages

**Figure 2:**
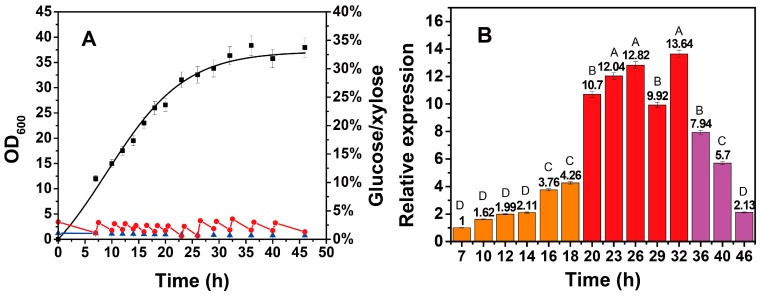
Growth and sugar consumption curves (**A**) and relative transcription of the xylose operon (**B**) in *B. licheniformis* during the fermentation process. In Figure 2A, the growth curve was obtained using a nonlinear fitting. In Figure 2B, Period I (7–20 h) was considered to be the logarithmic phase; Period II (20–36 h) was considered to be the stationary phase; and Period III (36–46 h) was considered to be the senescent phase. ■—■, OD_600_; ●—●, glucose concentration; ▲—▲, xylose concentration. In Figure 2B, ▄, first stage; ▄, second stage; ▄, third stage. Results are expressed as mean of replicates, one-way ANOVA followed by a Tukey’s post hoc test statistical analyses were performed. Treatments with different letters (where A > B > C > D) are significantly different at *p* < 0.01.

As the growth of cells globally and efficiently modulates the expression of all endogenous genes, the dynamic transcription of the xylose operon at different growth stages was first investigated. The cultivation was performed according to the above conditions, in which the glucose concentration was controlled at approximately 20 g/L. As shown in Figure 2A, the total consumption of xylose was 4.39 g/L until 46 h, and the bacterial growth was consistent with that shown in Figure 1, indicating a high degree of reproducibility. The transcription of the xylose operon obviously exhibited a three-period pattern based on the significantly different transcriptional activity (*n* = 3, *p* < 0.01) in Figure 2B. The logarithmic phase can be recognized as Period I (7–20 h), during which the rate of cell growth was the fastest, while the transcriptional activity of the xylose operon was relatively low. Period II (20–36 h) coincided with the stationary phase, during which the rate of cell growth started to decrease, and the transcriptional activity of the xylose operon sharply increased from 4.26 to 10.70, with a peak at 32 h. Period III (36–46 h) was the senescent phase, during which cell viability and the transcriptional activity of the xylose operon started to gradually decrease. Interestingly, the xylose operon still maintained an even higher expression strength within aging cells compared with that of the vigorous Period I. Also noteworthy is that the transcription of the xylose operon exhibited a significant upregulation during the transition from logarithmic phase to stationary phase (*n* = 3, *p* < 0.001), showing a distinct growth-phase-dependent expression pattern. In contrast, most promoters were observed to possess an analogous pattern with cell growth in *Bacillus subtilis* [26,27]. This nonsynchronism between cell growth and gene transcription may be attributed to the repressor protein XylR in the xylose operon, whose interaction with the promoter or with RNA polymerases is influenced by the growth phase, according to Harayama’s study [27]. Undoubtedly, this pattern is more conducive for separating cell-growth and protein-production stages from an application perspective [28,29], which is promising for the flexible and dynamic fine tuning of genes. Moreover, this result suggests that the xylose-inducible system in *B. licheniformis* is optimally induced at the beginning of the stationary phase, a period of crucial importance to achieve a high level of production during fermentation

### 2.3. Transcriptional Activity of the Xylose Operon under Glucose Stress

Glucose, as a competitive analogue to xylose, is presumed to be the most influential factor triggering the regulation of the xylose operon [30,31]. However, the degree of inhibition conferred by various glucose concentrations is not clear. We then explored the glucose-mediated CCR on the xylose operon in *B. licheniformis* at glucose concentrations ranging from 20 to 200 g/L. All the experimental groups were cultivated under the above standard conditions, and then glucose was added at different concentrations after 10 h to ensure that the effect of glucose stress on the transcriptional activity of the xylose operon could be studied independently of the effect of the growth stages. Time points 11 h, 31 h, and 45 h were chosen to represent each period (I, II, and III) for the transcription assay. The cell growth course and glucose and xylose concentrations at different time points are illustrated in Figure S2. Although the strain could survive in 200 g/L glucose, significant growth retardation was observed when it reached 180 g/L or more. Specifically, when cultivated in 200 g/L glucose, the cells did not enter the stationary phase until 30 h, which was over 5 h later than that in 20 g/L.

**Figure 3:**
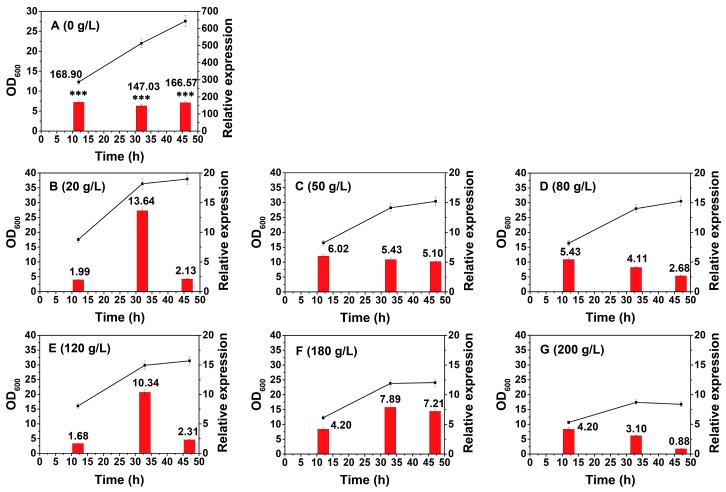
Growth and transcriptional activity of the xylose operon in *B. licheniformis* under glucose stress. ■—■, OD_600_, ▄, relative expression. The appropriate time point at each phase was selected to manually increase glucose concentration to the desired concentration during the fermentation process, with the transcriptional activity assessed after 1 h. The desired concentrations are as follows: **A**, 0 g/L; **B**, 20 g/L; **C**, 50 g/L; **D**, 80 g/L; **E**, 120 g/L; **F**, 180 g/L; **G**, 200 g/L. Results are expressed as mean of replicates, bars with *** represent statistically significant differences between groups at *p* < 0.001.

As for transcription, the most significant result is that 20 g/L glucose can even suppress the xylose operon to less than 10% compared with that in the glucose-free condition (Figure 3A,B, *n* = 3, *p* < 0.001). Within a glucose concentration range of 20–200 g/L, the relative expression level of each group merely changed over a small range. The suppression did not have an obvious enhancement with the increase in glucose (*n* = 3, *p* > 0.05), which suggested that the inhibition had reached saturation under 20 g/L glucose and that this inhibition was the dominant factor in the function of the xylose operon even under the conditions of huge differences in cell growth. The above results coincided with those of Hillen’s and Scott’s studies on the xylose operon from other bacterial strains, in which the inhibitory effect could reach 7–11-fold at glucose concentrations identical to those used in this study [32,33]. Another noteworthy observation is that the lowest transcription level was also detected under 200 g/L glucose. High concentrations of glucose exhibited a double inhibition of both cell growth and expression. The effect of glucose on growth retardation was previously illustrated as a response that occurs when high osmotic pressure leads to water efflux from microbial cells. It then causes a reduction in turgor and eventually leads to cessation of growth [34], resulting in globally negative effects on gene expression [35]. Overall, a further reduction in glucose concentration is required to minimize the negative effect on the expression of the operon. When using the xylose operon as an efficient expression element in *B. licheniformis*, the addition of a moderate amount glucose can be used for early cell growth, while dextrin or starch can be added for the subsequent expression of the target gene. As it is unrealistic to manually control the concentration to be much lower than 20 g/L during the fermentation process, a series of the amylase produced from the bacteria will slowly degrade dextrin or starch, producing a low-glucose environment to promote expression of the target product [35].

### 2.4. Transcriptional Activity of the Xylose Operon at Different Temperatures

**Figure 4:**
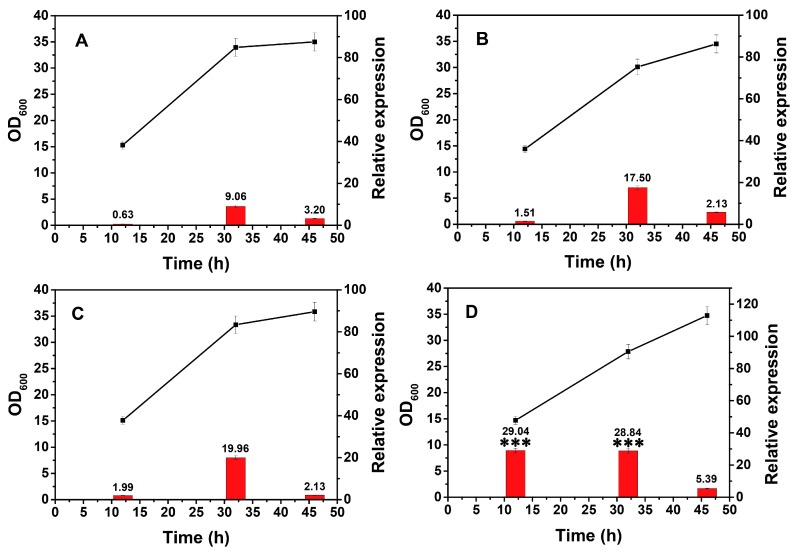
Relative transcription of the xylose operon in *B. licheniformis* at different temperatures. The cultures were transferred to shakers at different temperatures after being cultured to the desired time point with the transcriptional activity subsequently assessed after 1 h. ■—■, OD_600_, ▄, relative expression. **A**, 25 °C; **B**, 30 °C; **C**, 37 °C; **D**, 42 °C. Results are expressed as mean of replicates, bars with *** represent statistically significant differences between groups at *p* < 0.01.

Environmental temperature has direct effects on several fundamental biological processes. Thus, the influence of temperature on the performance of the xylose operon needs to be clarified. This experiment was similar to the glucose stress investigation: to eliminate the effect of different temperatures on the growth of the strain, which would globally influence the innate transcription activities. All the cultures were first incubated under the same temperature, 37 °C, then they were transferred to different temperature conditions. As predicted, the biomass of the precultures was essentially identical (Table S1). All the flasks were transferred into different temperatures (25, 30, 37, and 42 °C), and no significant differences in biomass of each culture could be detected after one hour (Table S1, *n* = 3, *p* > 0.05). This result ensured that the change in transcriptional activity was primarily caused by variations in temperature. As shown in Figure 4, the transcriptional activity at 25, 30, and 37 °C went through a similar process in which the initial transcription level (Period I) was weak and then increased dramatically in Period II. These trends agreed with the fact that the expression of this operon was dependent on the growth phase and reached its maximum during Period II.

In contrast, it reached a relative expression value of 29.04 in Period I and maintained this value in Period II at 42 °C, which was much higher than that under other temperatures (*n* = 3, *p* < 0.01). In particular, the value at 12 h was approximately 15-fold greater than that measured at 37 °C, and the 32 h value at 42 °C was still 1.5-fold greater than that observed at 37 °C (Figure 4). These results indicated an increased expression of the xylose operon from 25 to 42 °C. As previously described [36], many putative genes of regulons (i.e., HrcA, CtsR, and Sig B) showed elevated transcription when *B. licheniformis* cells reacted to a temperature elevation. The transcription of many operons is controlled by an RNA polymerase associated with a specific sigma factor, so the elevated transcription of the xylose operon at elevated temperatures may be associated with these regulons. *B. licheniformis* is a thermophilic platform, and several products (i.e., nattokinase, D-2,3-butanediol, and L-lactic acid) are expressed at 39–50 °C [37,38]. To a certain extent, this temperature-dependent expression behavior of *B. licheniformis* may have evolved in response to long-term growth in arid environments. It can be inferred that increasing the fermentation temperature within a certain range is an alternative strategy to improve the expression of the xylose operon.

### 2.5. Transcriptional Activity of the Xylose Operon at Different pH Values

**Figure 5:**
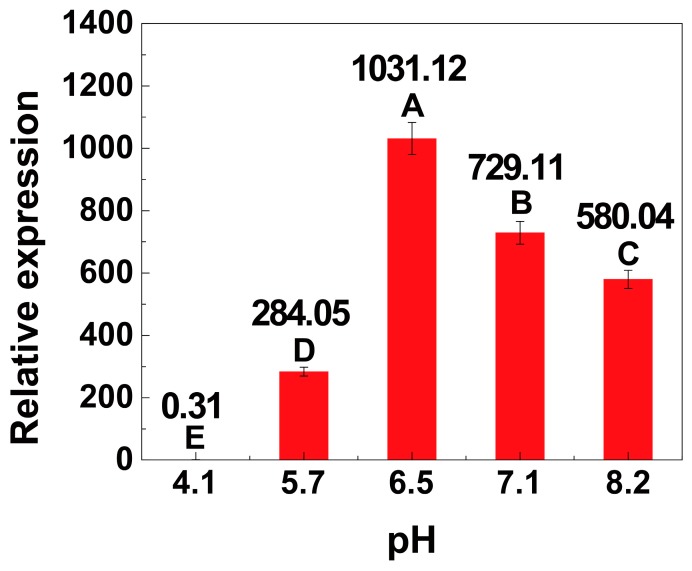
Relative transcription of the xylose operon in *B. licheniformis* at different pH values. Results are expressed as mean of replicates, one-way ANOVA followed by a Tukey’s post hoc test statistical analyses were performed. Treatments with different letters (where A > B > C > D > E) are significantly different at *p* < 0.01.

Finally, the effect of pH on the expression of the xylose operon was investigated to achieve a more comprehensive understanding of the xylose operon against environmental factors. As the pH is maintained constant during most industrial fermentation processes, its effect on cell growth at different stages is less important. As shown in Table 1 and Table S2, the pH of the groups was highly consistent (approximately 6.8) after 8 h of cultivation under the same conditions. When the concentrated buffers were added, the pH of the cultures was adjusted to different values close to expectations. After 1 additional hour of incubation, the pH had hardly changed, which guaranteed that the transcription responded to certain set pH values. The transcriptional activity at 9 h was calculated using the 4 h activity as a standard. As clearly shown in Figure 5, a significantly high level of transcriptional activity was observed when the pH was controlled between 6.5 and 7.1 (*n* = 3, *p* < 0.01). Specifically, the transcriptional activity at 9 h was only 0.31-fold that observed at 4 h at a pH of 4.12, while the transcriptional activity observed at 9 h was 1031.12- and 729.11-fold greater than that observed at 4 h at a pH of 6.50 and 7.14, respectively.

Although the pH was maintained at a set value, this value was slightly different from our desired pH. This slight difference did not influence the analysis of the characteristics of the xylose operon. 8^a^, pH before adjustment at 8 h; 8^b^, pH after adjustment at 8 h. The desired pH values are as follows: A, pH 4.00; B, pH 5.50; C, pH 6.50; D, pH 7.00; E, pH 8.00. Results are expressed as mean of replicates.

The role of pH during the fermentation of *B. licheniformis* has been previously demonstrated, revealing that the TCA (tricarboxylic acid) cycle, amino acid synthesis, glycolysis pathway, and enzyme synthesis are all affected by pH [39]. Thus, changes in the pH will inevitably lead to changes in gene transcription. As revealed in this assay, once the pH during fermentation decreased to less than 4, the cell growth and transcription of the xylose operon were both severely inhibited. It is thus conceivable that the growth of the cells and the transcription of the xylose operon would also be inhibited when the pH is higher than 8. Therefore, these results suggest that a neutral environment is essential to maintaining steady metabolic fluxes during fermentation to further improve the expression of the xylose operon.

### 2.6. Bioprocess of Recombinant B. licheniformis Based on Transcriptional Analysis

**Figure 6:**
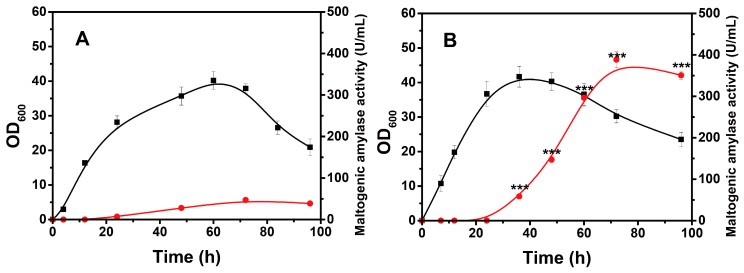
Growth and enzyme production curves of recombinant *B. licheniformis* strain under the unoptimized conditions (**A**) and optimized conditions (**B**). In Figure 6A, recombinant *B. licheniformis* strains were induced at 12 h, while in Figure 6B, the induction occurred at 24 h. In Figure 6B, pH values of 6.171, 6.689, and 7.706 were observed at 36, 60, and 96 h, respectively. ■—■, OD_600_; ●—●, *** represent statistically significant differences in maltogenic amylase activities between groups at *p* < 0.001.

The optimal induction time, carbon resource type, temperature, and pH were revealed based on the xylose operon transcriptional information. However, when this operon was used as a promoter element, its effects on the gene expression product in the actual fermentation process remained undefined. For this purpose, a recombinant *B. licheniformis* was constructed using maltogenic amylase as a reporter gene for assessment. The vector construction details and sodium dodecyl sulfate–polyacrylamide gel electrophoresis (SDS-PAGE) analyses are presented in Figure S3. The control group was performed according to the reported fermentation conditions in *Bacillus* strains, in which the expression was induced at the beginning of the logarithmic phase [40], with the addition of 20 g/L glucose and 10 g/L xylose every 24 h until 72 h (Figure 6A). Under these conditions, a maximum activity of only 47 U/mL was observed. In contrast, the expression was induced at the beginning of the stationary phase, where an initial 30 g/L glucose was added for the early cell growth and an initial 60 g/L dextrin was added as the later carbon source for the expression of the target gene (Figure 6B). As expected, a large amount of enzyme was generated during the stationary phase, and the maximum activity (388.5 U/mL) was significantly improved, which was approximately 8-fold greater than that of the control group (*p* = 0.01). Interestingly, using dextrin instead of glucose not only relieved the CCR effect but also contributed to maintaining the pH between 6.0 and 7.2 during the enzyme-producing stage. It is well known that abundant organic acids can be produced when bacterial cells utilize glucose rapidly. Dextrin, however, can be used at a much lower rate, which significantly limits acid accumulation [41,42]. Thus, the acid could be neutralized by alkaline substances synthesized during the enzyme-production phase, and the pH was maintained over a specific range.

In summary, the observed improvement in target-protein activity was caused by a combination of the optimal induction time, carbon source, temperature, and pH, indicating that transcription-based strategies are effective. Although many optimization studies have been reported [43,44,45], most of these focused on the amount of product rather than the fundamental law of the expression system. This causes one published strategy to rarely apply to other products, even if the same expression system is used. For example, we previously reported that a heterologous trehalose synthase (encoded by the gene *treS*) could be efficiently produced using a xylose-inducible system when the carbon source was maltodextrin and the induction time was at the end of the logarithmic phase, which agrees with the results in this study [46]. It is worth noting that the optimal temperature for that enzyme production was 37 °C, which was mainly determined by enzyme stability. Higher temperature (42 °C), although it would have a beneficial effect on transcriptional level, may damage the enzyme activity severely (Figure S4). It is easy to assume that the xylose operon transcription information provides the precondition for process optimization, and the strategies can be further fine-tuned according to the features of the product itself. Thus, avenues for future research are deep investigations of the post-transcriptional processes involving translation, modification, secretion, and so forth. Although slight differences between the transcription and expression occur, it is reasonable to make use of the proposed strategies to achieve efficient heterologous expression of genes of interest.

## 3. Materials and Methods

### 3.1. Strains, Plasmids, and Culture Conditions

The strains and plasmids used in this study are listed in Table 2. *B. licheniformis* ATCC 9945a was obtained from the Culture and Information Center of Industrial Microorganisms of China Universities (Wuxi, China) and used for transcriptional studies. *B. licheniformis* GM2, a *B. licheniformis* ATCC 9945a derivative that lacks amylase (encoded by *amyL*) and protease (encoded by *aprE*), served as an expression host.

Fifteen milliliters of LBG medium (lysogenic broth with 10 g/L glucose from Sinopharm Chemical, Shanghai, China) was used to activate a seed culture stored in a glycerol stock. Cultivation was performed in a 50 mL Erlenmeyer flask at 37 °C with shaking on a XT 5518 rotary shaker (Xutemp, Hangzhou, China) at 250 rpm. Fermentation medium (containing 20 g/L peptone, 10 g/L yeast extract, 5 g/L corn steep powder, 9.12 g/L K_2_HPO_4_, 1.36 g/L K_2_HPO_4_, 0.5 g/L CaCl_2_, and 0.5 g/L MgSO_4_·7H_2_O) was inoculated with 3% (*w*/*v*) of the activated seed culture, and then 30 mL of the medium was transferred to a 250 mL Erlenmeyer flask and cultivated at 37 °C with shaking on a rotary shaker at 250 rpm. Glucose, xylose (Sinopharm Chemical, Shanghai, China), and corn steep powder (Sinopharm Chemical, Shanghai, China) were autoclaved separately and then aseptically added to the medium as a concentrated solution.

### 3.2. Development of RT-qPCR Methods for B. licheniformis Fermentation

#### 3.2.1. Establishment of Culture Conditions

To develop RT-qPCR methods for fermentation analysis, the growth and metabolism of *B. licheniformis* must remain relatively stable, so basal culture conditions were established. Samples were taken from fermentation broth every 2–4 h. Subsequently, the residual glucose concentration was immediately detected using the dinitrosalicylic acid (DNS, Sinopharm Chemical, Shanghai, China) method to avoid the glucose that was used up by the strain. Then, glucose was continuously added to the fermentation medium to maintain the glucose concentration at approximately 20 g/L throughout the fermentation process. The cell density was measured simultaneously.

#### 3.2.2. Investigation of Transcriptional Activity at Different Growth Stages

To investigate the transcriptional activity of the xylose operon at different growth stages, 10 g/L xylose was added at the onset of fermentation to induce *xylA* gene expression. All other culture conditions were as established above. Samples were collected at every interval, and the aliquots were stored at −70 °C. Residual glucose and cell density were detected as described above.

#### 3.2.3. Investigation of Transcriptional Activity under Glucose Stress

To investigate the transcriptional activity of the xylose operon under glucose stress, the experiment was performed in two stages based on previous results. In the first stage, the *Bacillus* strain was inoculated in six replicates of fermentation medium containing 30 g/L glucose and 10 g/L xylose and cultured at 37 °C for 10 h. In the second stage, glucose was added at varying concentrations (20, 50, 80, 120, 180, and 200 g/L). For the set of glucose starvation, the initial carbon resources were also 30 g/L glucose and 10 g/L xylose. After the glucose was completely consumed, 20 g/L xylose was fed at 11, 31, 42, and 45 h to ensure the activation of the transcription. The respective cell culture samples were collected after 1 h, which ensured that the operon had adapted to the glucose concentration. The samples were immediately subjected to RNA extraction and subsequent RT-qPCR assay.

#### 3.2.4. Investigation of Transcriptional Activity at Different Temperatures

The *Bacillus* strain was inoculated in the same fermentation media and cultivated according to the above established methods. After 10 h, the cultures were transferred to different temperatures ranging from 25 to 42 °C and supplemented with 20 g/L xylose. The cell culture samples were collected after 1 h and immediately subjected to RNA extraction and subsequent RT-qPCR assay.

#### 3.2.5. Investigating Transcriptional Activity at Different pH Values

The *Bacillus* strain was inoculated in the same fermentation media and cultivated according to the above established methods. After 10 h, the pH was adjusted with a 2 mol/L ammonia solution or 2 mol/L sulfuric acid to the desired pH values of 4.0, 5.5, 6.5, 7.0, and 8.0. Simultaneously, 20 g/L xylose was added to the medium to induce *xylA* expression. Cell density and pH were measured after another 1 h of cultivation, and samples were immediately subjected to RNA extraction and subsequent RT-qPCR assay.

### 3.3. Relative Quantitative Real-Time PCR (RT-qPCR)

All of the RT-qPCR procedures were performed in accordance with the MIQE guidelines [47]. First, the samples stored at −70 °C were thawed in an ice bath. Subsequently, total RNA was extracted from the samples using TIANGEN reagent (TIANGEN, Beijing, China) according to the manufacturer’s protocol, and the RNA concentrations were determined using a Quawell Q5000 ultraviolet-visible spectrophotometer (Quawell, San Jose, CA, USA). The results of 2% agarose gel electrophoresis (see the Supplementary Material) and the ratio of the absorbances (2.1 ± 0.5) at 260 nm and 280 nm indicated that the RNA purity was acceptable. Subsequently, cDNA was amplified from approximately 500 ng of total RNA using a TaKaRa kit (TaKaRa, Osaka, Japan) and further diluted to 200 ± 50 ng/μL for use as a template for RT-qPCR. Finally, RT-qPCR was performed in 10 μL mixtures in triplicate using a Bio-Rad CFX96 real-time PCR detection system (Bio-Rad Laboratories, Hercules, CA, USA). The mixture for one reaction contained 5 µL of 1× SYBR Green Master Mix, 0.4 µL of each primer at 20 μmol/L, and 0.5 μL of cDNA template. RT-qPCR was achieved using the following conditions: 95 °C for 30 s and 40 cycles of 95 °C for 5 s and by 60 °C for 30 s, after which the fluorescent signal was detected.

The 2^−ΔΔ*C*t^ method was used to calculate the relative changes in *xylA* gene expression levels between various fermentation conditions [48]. Primer pairs (rpsE1/2 and xylA1/2) for *rpsE* and *xylA* amplification (see the Supplementary Material) were designed based on gene sequences obtained from the National Center for Biotechnology Information (NCBI). The RT-qPCR amplification efficiencies were calculated using the formula $E=(10^{-1/s}-1)\times 100\%$, where s is the slope of the standard curve with several dilutions of cDNA [49]. The expression of the *xylA* gene was determined relative to that of *rpsE* [50,51] by RT-qPCR.

### 3.4. Construction of Xylose-Inducible Expression Strains and Fermentation

A series of *E. coli*/*Bacillus* shuttle vectors were constructed based on the plasmid pHY300-PLK. The PCR primers used in this experiment are listed in Table S3. First, *B. licheniformis* 9945a genomic DNA and the plasmid pBSSY harbored by *E. coli* JM109 were extracted and used as a template for PCR. The xylose regulon (*blxyl*) encoding the xylose promoter and repressor was amplified from the genomic DNA, a fusion fragment (*sacBss*- *yvdF*- *ter*) consisting of the maltogenic amylase gene and the terminator sequence from *B. licheniformis* ATCC 14580, and a levansucrase signal peptide sequence from *B. subtilis* 168 was amplified from the plasmid pBSSY. PCR amplification was performed using Pfu polymerase (TaKaRa, Osaka, Japan) and the primer pairs Blxyl F/R and BLMA F/R. Subsequently, the DNA fragments and the plasmid pHY300-PLK were digested with the respective restriction enzymes, and all of the fragments were cloned into pHY300-PLK, a xylose-inducible vector that was designated pBLSY. Finally, pBLSY was transferred into *B. licheniformis* GM2 using the method described by Li [8].

A 60 mL primary culture (fermentation medium, 20 µg/mL tetracycline from Sinopharm Chemical, Shanghai, China) was inoculated with 1.8 mL of the starter culture and grown in 250 mL three-baffle Erlenmeyer flasks at 37 °C with shaking at 250 rpm. In addition, 10 g/L diammonium hydrogen phosphate (Sinopharm Chemical, Shanghai, China) was added to the medium as an early nitrogen source.

### 3.5. Analytical Procedures

#### 3.5.1. Cell Density Determination

Fermentation samples were suitably diluted to determine the cell density by measuring the absorbance at 600 nm with a UV-Vis spectrophotometer.

#### 3.5.2. Sugar Content Analysis during Fermentation

The sugar content analysis was mainly performed in two methods. The reducing sugar concentration was detected during the fermentation process using the DNS method [52], because it is simple and fast, and the results could be immediately taken into consideration for carbon source feeding, although the accuracy of this method is limited. For precise quantification of xylose or glucose, a high-performance liquid chromatography (HPLC) instrument (Dionex, Sunnyvale, CA, USA) equipped with a sugar separation column (Shodex SH1011, Tokyo, Japan) was used. The samples were centrifuged at 12,000 *g* for 10 min, and the supernatants were stored at 4 °C. After the culture was completed, samples were pretreated for protein removal and then subjected to HPLC analysis. Ten mM H_2_SO_4_ (0.8 mL/min) was used as the mobile phase at 50 °C, and glucose and xylose were detected using a differential refractometer [23]. A standard curve was generated using D-glucose and D-xylose solutions.

#### 3.5.3. Enzyme Assays

Extracellular maltogenic amylase activities were determined using the method described by Li [9].

#### 3.5.4. Statistical Analysis

All experiments were performed at least three times, and the results were expressed as the means ± standard deviations (SDs). Statistical analyses were performed using Student’s *t* test and ANOVA followed by a Tukey’s post hoc test. P values of less than 0.05 indicated significant results.

## 4. Conclusions

The effect of various fermentation conditions on the transcriptional activity of the xylose operon in B. licheniformis was investigated. High transcriptional activity was observed in the stationary phase (approximately 2–10-fold that observed in the logarithmic phase). The inhibitory effect of glucose hardly strengthened at concentrations ranging from 20 to 180 g/L. Furthermore, the maintenance of a higher temperature (42 °C) and a neutral pH (pH 6.5–7.0) during the fermentation process increased the transcriptional level. Based on these features, maltogenic amylase was used as a reporter protein to verify the relevant strategies. As a result, the observed recombinant enzyme activity was approximately 8-fold greater than that observed under unoptimized conditions in induction time, carbon resource type, and pH. The only incompatible condition was temperature, as a lower one was beneficial for protection of enzyme activity. The results of our study provide novel insights into fermentation optimization.

## Figures and Tables

**Table 1:** Changes in pH after 1 h at different desired pH values.

Time (h)	pH Values				
	A	B	C	D	E
8 *	6.84 _a_	6.82 _a_	6.86 _a_	6.83 _a_	6.87 _a_
8 **	4.15 _e_	5.71 _d_	6.51 _c_	7.14 _b_	8.20 _a_
9	4.12 _e_	5.67 _d_	6.35 _c_	6.94 _b_	7.93 _a_

* Before concentrated pH buffers were added; ** after concentrated pH buffers were added. Results are expressed as mean of three replicates. Statistical analyses of A, B, C, D, E were considered independently: a–e, means with different lower-case letters for pH values are significantly different (ANOVA; *p* < 0.05).

**Table 2:** Bacterial strains and plasmids.

Strain or Plasmid	Description	Source/Reference
*Escherichia coli* JM109	*endA1, recA1, gyrA96, thi, hsdR17 (rk−, mk+), relA1, supE44, Δ( lac-proAB), [F′ traD36, proAB, laqIqZΔM15]*	CICIM-CU ^1^
*Bacillus licheniformis* 9945a	Wild-type strain	CICIM-CU
*Bacillus licheniformis* GM2	*Bacillus licheniformis* 9945a derivative, deficient in Δ*amyL*, Δ*aprE*	GY. Shi, this lab
pHY300-PLK	*E.coli*/*Bacillus* shuttle vector, Ap^R^/Tet^R^	TaKaRa
pBLxyl	pHY300-PLK derivative with the xylose regulon from *B. licheniformis* 9945a	This work
pBLSY	pHY300-PLK derivative with the xylose regulon from *B. licheniformis* 9945a, the signal peptide from the levansucrase-encoding gene of *B. subtilis* 168 (*sacBss*), and the maltogenic amylase (*yvdF*)-encoding gene and terminator sequence from *B. licheniformis* DSM13	This work
pBSSY	pHY300-PLK derivative with the xylose regulon from *B. subtilis* 168, the signal peptide from the levansucrase encoding gene of *B. subtilis* 168 (*sacBss*), and the maltogenic amylase (*yvdF*)-encoding gene and terminator sequence from *B. licheniformis* DSM13	GY. Shi, this lab

^1^ CICIM-CU, Culture and Information Center of Industrial Microorganisms of China Universities; Ap^R^, ampicillin resistance; Tet^R^, tetracycline resistance.

## Supplementary Materials

Supplementary materials can be found at https://www.mdpi.com/1422-0067/20/18/4615/s1.
