# Supplementary material for: Transcriptional Changes in the Xylose Operon in Bacillus licheniformis and Their Use in Fermentation Optimization
Source: Int J Mol Sci. 2019 Sep 18;20(18):4615. doi: 10.3390/ijms20184615 (PMC6769896; doi:10.3390/ijms20184615)
Supplement: Supplementary file 1 [file ijms-20-04615-s001.pdf]

Supplementary

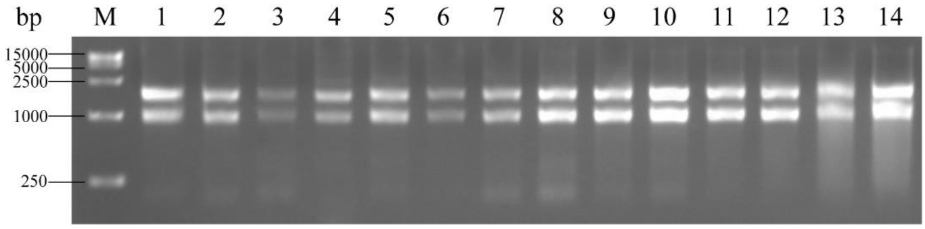

Figure S1. Total RNA extracted from samples at different time points. 1-14 are RNAs extracted from the samples during different stages of the fermentation process, and analyzed using a 2% agarose gel electrophoresis. The electrophoresis strips from top to bottom of 1-14 are: 23S rRNA, 16S rRNA, 5S rRNA.

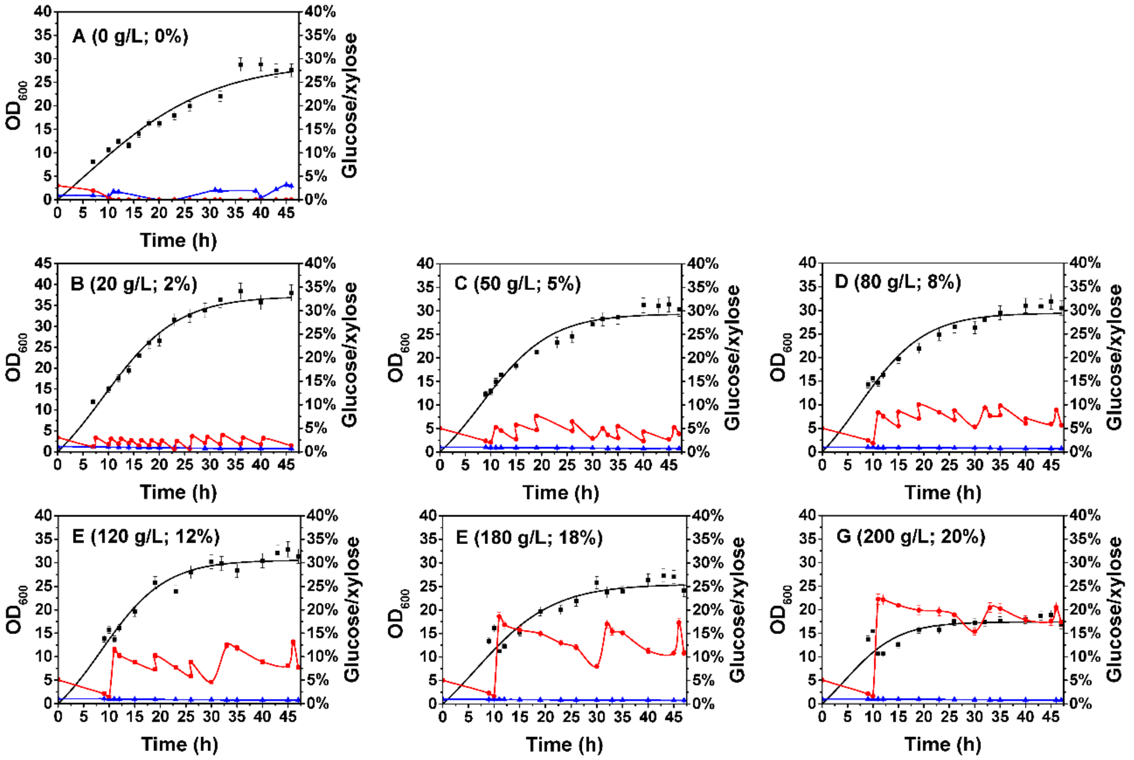

Figure S2. Growth and sugar consumption curves of *B.licheniformis* under glucose stress. ■—■, OD_{600}; ●—●, glucose concentration; ▲—▲, xylose concentration. **A**, 0 g/L (0%); **B**, 20 g/L (2%); **C**, 50 g/L (5%); **D**, 80 g/L (8%); **E**, 120 g/L (12%); **F**, 180 g/L (18%); **G**, 200 g/L (20%). Where the growth curve is obtained using a nonlinear fitting. The decrease of the biomass in the growth is due to the dilution of the fermentation broth by the supplemented glucose.

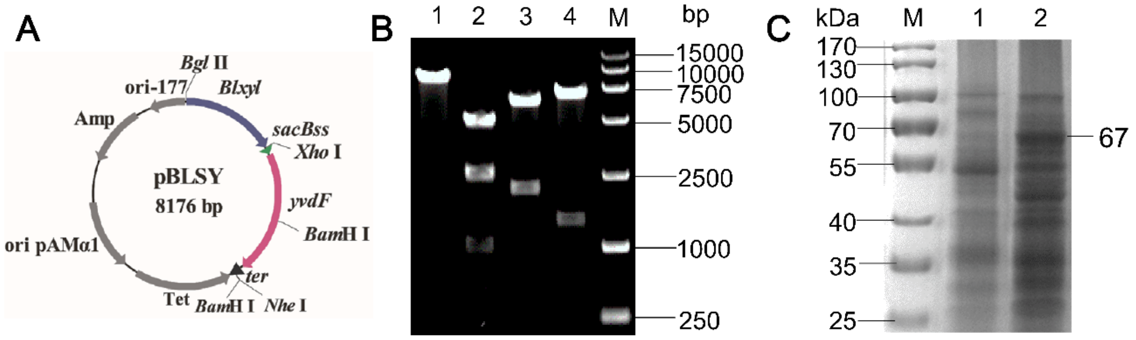

Figure S3. Map of plasmid pBLSY (**A**), restriction pattern of pBLSY (**B**) and SDS-PAGE chromatogram of the crude enzyme produced by recombinant B. licheniformis GM2 (**C**). In Figure. S3 (**B**), M, lambda DNA Maker; 1, pBLSY/Bgl II; 2, pBLSY/Bgl II + BamH I; 3, pBLSY/Xho I+Nhe I; 4, pBLSY/Bgl II + Xho I. In Figure. S3 (**C**), M, protein maker; 1, extracellular crude enzyme after uninducing; 2, extracellular crude enzyme after inducing.

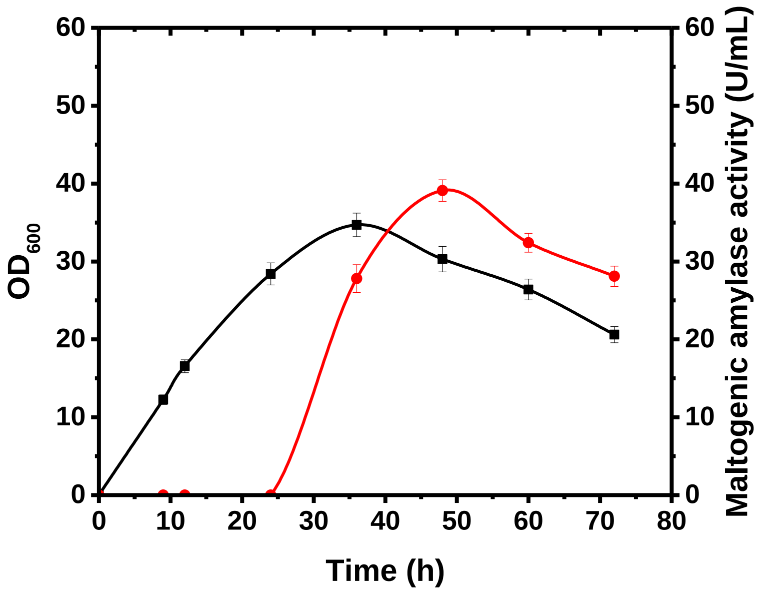

Figure S4. Growth and enzyme production curves of recombinant *B. licheniformis* strain at 42℃. ■—■, OD_{600}; ●—●, maltogenic amylase activity. Extracellular protein concentration of 0.55, 0.59, 0.64, 0.68 g/L were observed at 36, 48, 60, 72 h, respectively.

Table S1. Changes of biomass after one-hour cultivation at different temperature shocks.

Sampling Time (h)	Biomass (OD_{600} at different desired temperatures)			
	A	B	C	D
11	14.45 $_a$	14.25 $_a$	14.50 $_a$	14.30 $_a$
12	15.30 $_a$	14.40 $_b$	15.10 $_a$	14.70 $_b$
31	33.10 $_a$	29.25 $_b$	32.55 $_a$	27.00 $_b$
32	33.95 $_a$	30.10 $_b$	33.35 $_a$	27.85 $_c$
45	34.40 $_a$	34.75 $_a$	35.10 $_a$	34.55 $_a$
46	35.00 $_a$	34.50 $_a$	35.85 $_a$	34.75 $_a$

A, 25℃; **B**, 30℃; **C**, 37℃; **D**, 42℃. Results are expressed as mean of 3 replicates. Statistical analyses of A, B, C, D were considered independently): a–c—Means with different lower-case letters for pH values are significantly different (ANOVA; $p < 0.05$).

Table S2. Changes of biomass after one-hour cultivation at different pH shocks.

Sampling Time (h)	Biomass (OD_{600} at different desired pH)				
	A	B	C	D	E
4	4.38 $_c$	4.32 $_c$	4.78 $_b$	5.24 $_a$	5.04 $_b$
8	7.86 $_b$	7.26 $_c$	7.85 $_b$	8.69 $_a$	7.35 $_c$
9	7.71 $_b$	7.08 $_c$	8.24 $_b$	9.24 $_a$	7.65 $_b$

A, pH 4.00; B, pH 5.50; C, pH 6.50; D, pH 8.00. Results are expressed as mean of 3 replicates. Statistical analyses of A, B, C, D, E were considered independently): a–c—Means with different lower-case letters for pH values are significantly different (ANOVA; $p < 0.05$).

Table S3. List of primers used for vector construction and qPCR analysis.

Primer name.	Sequence(5′-3′)$^{\alpha}$	restriction sites

Blxyl F	GGAAGATCTTTAAAATCTCTCGTTCATAAACCGTTCCA GAAAACGTTCGGTTACAAGGG	*Bgl* II
Blxyl R	TTCGCGGATCCCTGCTAGCCCGCTCGAGTCCGATCTC CCCCTTCACTTTT	*Xho* I，*Nhe* I，*Bam*H I
BLMA F	CCGCTCGAGATGAACATCAAAAAGTTTGC	*Xho* I
BLMA R	CTAGCTAGCGGGTAAAAAACCATTCACTC	*Nhe* I
RpsE1	TGGTCGTCGTTTCCGCTTCG	
RpsE2	TCGCTTCTGGTACTTCTTGTGCTT	
XylA1	CGAAAGAGCCGACGAAGCA	
XylA2	ATCCCAGCCCAGGAGCAAA	

[α]Restriction sites are underlined.

Appendix A

The explanation to E. coli JM109 genotype

endA1 glnV44 thi-1 relA1 gyrA96 recA1 mcrB+ Δ(lac-proAB) e14- [F' traD36 proAB+ lacIq lacZΔM15] hsdR17(rK -mK +)

endA1: This strain lacks Endonuclease I (non-specific digestion) for cleaner preparations of DNA and better results in downstream applications.

glnV44: In this strain a suppression of amber (UAG) stop codons (required for some phage growth) by insertion of glutamine was carried out.

thi-1: This strain requires thiamine (thiamine auxotroph, cannot produce its own thiamine).

relA1: *Escherichia coli* (*relA1*) develop a lipid structure that radically differs from the wild type and is characterized by accumulation of neutral phospholipids and saturated fatty acids. The membrane is more fragile with respect to sonication and osmotic chock. Protein leakage and cell lysis is, however, lower in the mutant most likely due to the increased amounts of saturated fatty acids, which might be a possible strategy to overcome the reduced amounts of membrane-strengthening cardiolipinrelaxed phenotype; it also permits RNA synthesis in absence of protein synthesis. Ref: http://www.ncbi.nlm.nih.gov/pubmed/16718493

gyrA96: The strain has a mutation in DNA gyrase which conveys nalidixic acid resistance (the gyrase mutation *gyrA96* gives *E. coli* a ccdB resistance). Ref: http : // parts.igem.org / Part : BBa_P1010: Experience

recA1: *RecA* is a protein used by *E. coli* to repair and maintain DNA. RecA1 is an inactivated form of *RecA*. *RecA1* is deficient in all known function of the *RecA* gene specifically in ATPase activity, binding with DNA in the presence of ATP, and changing conformation in the presence of ATP and repressor cleavage. Cells of this genotype are UV sensitive due deficiencies in DNA repair mechanisms. Why select this mutation in *E. coli* lab strains? For reduced occurrence of unwanted recombination in cloned DNA. Partly restriction-deficient; good strain for cloning repetitive DNA (*RecA*–). Ref: http://2011.igem.org/RecA_Project

mcrB+: *McrB* (modified cytosine restriction) system of E. coli interferes with incoming DNA containing methylcytosine. DNA from many organisms, including all mammalian and plant DNA, is expected to be sensitive, and this could interfere with cloning experiments. Ref: http://www.ncbi.nlm.nih.gov/pubmed/2831502

Δ(lac-proAB): Deletion of the entire lac operon and also of the genes proA and proB that encode the first two enzymes of the proline biosynthetic sequence in Escherichia coli (so the cell requires proline for its growth).

e14-: Absence of the prophage like element containing *mcrA* gene (the *McrA* system, as the *McrB* described above, is involved in the restriction of DNA sequences containing methylated cytosine at particular sequences).

F'[…]: Host contains an F' episomal plasmid with the stated features.

¬ ***traD36***: Mutation eliminating transfer factor; prevents transfer of F plasmid. Ref: *E. coli* Genetic Resources at Yale. CGSC, The *Coli* Genetic Stock Center

¬ ***proA+B+***: The genes *proA* and *proB* encoding the first two enzymes of the proline biosynthetic sequence in *Escherichia coli*.

¬ ***lacIq***: Overproduction of the lac repressor protein (-35 site in promoter upstream of *lacI* is mutated from GCGCAA to GTGCAA).

¬ ***lacZΔM15***: This *E. coli* strain carries the *lacZ* deletion mutant which contains the ω-peptide: a mutant β-galactosidase derived from the M15 strain of *E. coli* that has its N-terminal residues 11—41 deleted and is unable to form a tetramer so it is inactive. The plasmids used in transformation process carry the *lacZα* sequence which encodes the first 59 residues of β-galactosidase (the *α*-peptide). Neither is functional by itself, however, when the two peptides are expressed together they form a functional β-galactosidase enzyme. Required for blue/white selection on XGal plates.

hsdR17(rK -mK +): The *hsdR17* mutation eliminates the restriction endonuclease of the restriction-modification system, *Eco*KI, so DNA lacking the *Eco*KI methylation will not be degraded (prevents cleavage of heterologous DNA by an endogenous endonuclease), but the corresponding methylase gene (*hsdM*) is present. To sum up, this strain is restriction deficient but still methylate's DNA.
